# Clinical and economic burden associated with symptomatic and asymptomatic obstructive hypertrophic cardiomyopathy in Germany

**DOI:** 10.1007/s00392-025-02776-4

**Published:** 2025-10-27

**Authors:** Farbod Sedaghat-Hamedani, Carla L. Zema, Michael Schultze, Tarcyane B. Garcia, Nils Kossack, Julia Borchert, Ervant J. Maksabedian Hernandez, Yue Zhong, Tobias Bluhmki, Taryn Krause, Johanna Schmoelders, Benjamin Meder

**Affiliations:** 1 Department of Internal Medicine III, Institute of Cardiomyopathies, University of Heidelberg, INF 410, 69120 Heidelberg, Germany; 2 DZHK (German Centre for Cardiovascular Research), Heidelberg, Germany; 3 Bristol Myers Squibb, Princeton, NJ USA; 4 ZEG – Berlin Center for Epidemiology and Health Research GmbH, Berlin, Germany; 5 WIG2 GmbH – Scientific Institute for Health Economics and Health System Research, Leipzig, Germany; 6 Bristol Myers Squibb, Munich, Germany; 7 Bristol Myers Squibb, Uxbridge, UK

**Keywords:** Cardiovascular outcomes, Clinical and economic burden of illness, Symptomatic and asymptomatic obstructive hypertrophic cardiomyopathy, Healthcare costs, Healthcare resource utilization

## Abstract

**Background:**

The clinical and economic burden of obstructive hypertrophic cardiomyopathy (HCM) in Germany for symptomatic versus asymptomatic patients has not been comprehensively assessed.

**Methods:**

This retrospective observational study analyzed nationally representative WIG2 Benchmark Database data, identifying adults diagnosed with obstructive HCM from 2012 to 2018. Study index was the first date when a participant met eligibility criteria. An algorithm based on coded symptoms and pharmacological treatments was used to divide patients into symptomatic and asymptomatic subgroups. Annual prevalence, patient characteristics, outcomes, resource utilization, and costs were assessed during follow-up.

**Results:**

Overall, 1141 patients were included (649 symptomatic patients and 492 asymptomatic patients during the 1-year pre-index period [baseline]). In total, 1042 patients had symptomatic disease at some point during follow-up. Annual obstructive HCM prevalence increased between 2011 and 2019 and was higher for symptomatic than asymptomatic patients. Compared with asymptomatic patients, symptomatic patients: were older (mean age: 62.8 vs. 55.5 years), were less likely to be male (59% vs. 67%), and had higher Charlson Comorbidity Index (mean: 2.82 vs. 1.69) at baseline; had higher rates of all-cause mortality (0.05 vs. 0.02 per 100 patient-years) and cardiovascular events (mean follow-up: 4.7 years); had more outpatient visits (13.3 vs. 10.8 per patient-year [PPY]), inpatient visits (0.7 vs. 0.5 PPY), and longer mean length of stay (8.7 vs. 7.4 days); and had higher mean costs PPY for outpatient, inpatient, and pharmacy services (total: €5975 vs. €4399).

**Conclusions:**

Patients with symptomatic obstructive HCM experienced greater clinical and economic burden than asymptomatic patients in Germany.

**Graphical Abstract:**

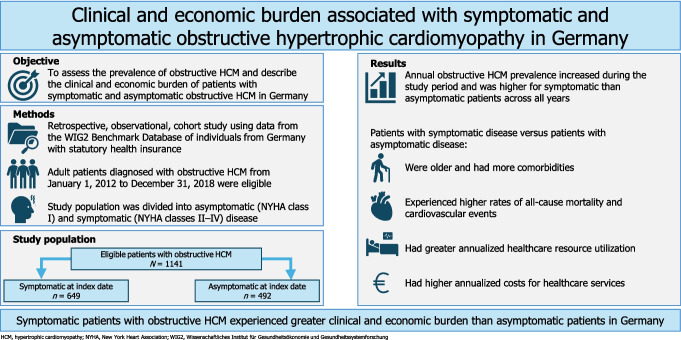

**Supplementary Information:**

The online version contains supplementary material available at 10.1007/s00392-025-02776-4.

## Introduction

Hypertrophic cardiomyopathy (HCM) is a myocardial disorder characterized by increased left ventricular wall thickness or mass that is not explained by abnormal loading conditions [1]. The estimated prevalence of HCM in the general population has been reported as 1:500 (0.2%) [2–5]. Prevalence estimates based on electronic health records and claims data vary, with previous studies reporting prevalences of 0.03% in the USA [6] and between 0.07% and 0.09% in Germany [7, 8]. It is estimated that up to 70% of patients with HCM have left ventricular outflow tract (LVOT) obstruction (defined as an LVOT gradient ≥ 30 mmHg at rest, or with physiological provocation) [9]. However, few studies have reported country-based prevalence estimates specifically for obstructive HCM [10].

Patients with obstructive HCM may remain asymptomatic, although some experience symptoms such as fatigue, dyspnea, chest pain, palpitations, and presyncope or syncope [1, 11]. An analysis based on electronic health records in the USA demonstrated that worsening symptom burden, as measured by New York Heart Association (NYHA) functional class, was associated with increased risks of all-cause mortality and cardiovascular outcomes in obstructive HCM [12]. Moreover, previous research in the USA has described a substantial clinical and economic burden associated with obstructive HCM [13]. In Germany, a study in patients with chronic systolic heart failure identified HCM as the most resource-intensive etiology, with higher resource utilization and costs compared with patients with other cardiac conditions, such as coronary artery disease [14].

Little evidence is available regarding the burden of obstructive HCM in Germany. This study aimed to assess the prevalence of obstructive HCM and describe the associated clinical and economic burden in patients with symptomatic and asymptomatic disease in Germany.

## Methods

### Data source and study design

This retrospective, observational, cohort study used data from the Wissenschaftliches Institut für Gesundheitsökonomie und Gesundheitssystemforschung (WIG2) Benchmark Database, a nationally representative healthcare claims database with longitudinal data for approximately 4.5 million individuals in Germany with statutory health insurance (SHI) from contributing insurance providers. Data in the WIG2 database are anonymized with respect to individual insurance, healthcare providers (e.g., physicians, practices, hospitals, pharmacies), and the respective SHI. The dataset was shown to be representative of the German SHI population for age, sex, and morbidity [15].

**Fig. 1:**
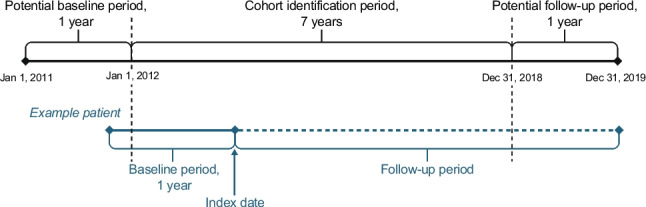
Study design. The blue line represents the timeline of an example patient included in the study

Patients with a diagnosis of obstructive HCM were identified from January 1, 2012 to December 31, 2018 (cohort identification period). The index date was defined as the beginning date of the first quarter in the cohort identification period when a participant had a diagnosis of obstructive HCM and met study eligibility criteria. The study comprised a pre-index period (‘baseline period’) of 1 year before the index date, and a follow-up period until the end of the study period (December 31, 2019), the date of death, or the date of the end of data availability in the database, whichever occurred first (Fig. 1).

### Study population

This study included patients with obstructive HCM, as identified by at least one diagnosis code in the International Classification of Diseases, 10th Revision, Germany Modification (ICD-10-GM) for obstructive HCM (ICD-10-GM I42.1) or for other HCM (ICD-10-GM I42.2 and/or I42.9) in combination with a code for septal reduction therapy (SRT). Eligible patients were adults (aged ≥ 18 years) at the time of diagnosis who had ≥ 1 year of data available before the index date (baseline period) and ≥ 1 year of follow-up data (except in the case of death) (Fig. 1). Patients with diagnostic codes indicating amyloidosis, aortic stenosis, hypertensive heart disease, or storage diseases were excluded.

The study population was divided into two subgroups: individuals with asymptomatic obstructive HCM and those with symptomatic obstructive HCM. The asymptomatic subgroup included participants in NYHA functional class I, and the symptomatic subgroup included participants with NYHA functional class II, III, or IV symptoms. NYHA functional class assignments were not documented in the source database; therefore, the classification was assigned based on a stepwise algorithm, developed with expert clinical input, that accounted for ICD-10-GM codes for heart failure, treatments used (for obstructive HCM, heart failure, or atrial fibrillation), and relevant symptoms (e.g., fatigue, palpitations, shortness of breath, chest pain). Results presented here were attributed to the subgroup in which the patient resided at the time of the documented outcome; for example, if a patient had asymptomatic disease at the index date, but was hospitalized after their disease became symptomatic, the hospitalization was attributed to the symptomatic subgroup. Therefore, patients with asymptomatic disease at the index date could contribute data to both subgroups.

### Outcomes

Prevalence of obstructive HCM was assessed for each calendar year from 2011 to 2019 (i.e., from the start of the earliest potential baseline period to the end of the study period). Data from the WIG2 database were used to extrapolate the prevalence of obstructive HCM in the SHI population. Patient characteristics were assessed at the index date, and comorbidities were evaluated during the baseline period. Health outcomes (all-cause mortality and cardiovascular events), healthcare resource utilization, and costs were analyzed during the follow-up period.

For health outcomes, the number of events per patient-year and the time from the index date to the first incidence of each condition/event were assessed. To account for differences in patient follow-up times, healthcare resource utilization and costs were annualized to allow comparison between the symptomatic and asymptomatic subgroups. In the outpatient setting, healthcare resource utilization was measured by the number of cases, representing care from the same provider for the same reason within a quarter, which could have included multiple visits. Data were described for the total population and across asymptomatic and symptomatic subgroups.

### Statistical analyses

Descriptive summary statistics were used to assess continuous variables. For discrete variables, the frequency and percentage for each category were assessed. Prevalence of HCM was estimated in the WIG2 database and extrapolated to the population size of all patients covered by SHI, using Statistik über Versicherte, gegliedert nach Status, Alter, Wohnort und Kassenart (KM6) statistics for calculating population size standardized by age and gender [16]. All statistical analyses were conducted using Microsoft SQL Server 2012 and R software version 3.2.3.

## Ethics approval

Given that all data in the WIG2 Benchmark Database are anonymized to comply with German privacy regulations, their use for scientific purposes is in conformity with German law, and no additional permission is needed from an institutional review board or an independent ethics committee. The nature of the study presents no risk to the respondents because data have already been anonymized and no patient can be identified from the analysis of secondary data; therefore, informed consent is not required.

## Results

In total, 6793 patients were identified with any HCM diagnosis, including 2922 with obstructive HCM. Overall, 1141 patients with obstructive HCM met the eligibility criteria and were included in the study (Supplementary Fig. S1). Of these patients, 649 had symptomatic disease and 492 had asymptomatic disease at index.

### Prevalence of obstructive HCM

Annual prevalence of obstructive HCM is summarized in Table 1. In the SHI population, a linear increasing annual trend in the prevalence of obstructive HCM was observed from 2012 to 2019. Annual prevalence ranged from 42.5 per 100,000 people (0.04%) in 2011 to 52.9 per 100,000 people (0.05%) in 2019.

**Table 1:** Prevalence of obstructive HCM per year in the WIG2 DB population and SHI population, 2011–2019

Year	Prevalence per 100,000 patients per year					
	Total obstructive HCM		Asymptomatic		Symptomatic	
	DB	SHI	DB	SHI	DB	SHI
2011	34.22	42.53	12.75	14.57	21.47	27.95
2012	34.66	42.83	12.48	14.42	22.17	28.41
2013	37.50	45.09	12.64	14.07	24.86	31.02
2014	40.56	47.96	14.10	15.04	26.46	32.92
2015	42.54	49.42	13.34	14.23	29.21	35.20
2016	43.72	49.70	13.81	14.66	29.91	35.04
2017	46.04	50.44	14.74	15.11	31.30	35.32
2018	48.28	51.69	15.84	15.98	32.44	35.70
2019	50.55	52.86	15.86	16.02	34.69	36.84

The values in the DB columns represent unadjusted data; these data were used to extrapolate the prevalence of obstructive HCM in the SHI population. *DB*, database; *HCM*, hypertrophic cardiomyopathy; *SHI*, statutory health insurance

The annual prevalence of obstructive HCM was higher in the symptomatic subgroup than in the asymptomatic subgroup across all years. Prevalence trends were similar for both, with a gradual increase in annual prevalence being observed from 2011 to 2019 (Table 1).

Age- and sex-stratified overall prevalence of obstructive HCM in the SHI population during 2019 is presented in Supplementary Fig. S2. Prevalence of obstructive HCM increased with increasing age in both men and women, with the lowest prevalence being among the 18–29-years age group (men: 11.1 per 100,000 person-years; women: 7.1 per 100,000 person-years) and the highest prevalence being observed for the ≥ 80-years age group (men: 142.0 per 100,000 person-years; women: 160.8 per 100,000 person-years). In both men and women, prevalence was consistently higher in the symptomatic subgroup than in the asymptomatic subgroup for all age groups ≥ 40 years. Prevalence of symptomatic and asymptomatic obstructive HCM generally increased with increasing age in men and women.

### Patient characteristics

Baseline patient characteristics, medical history, and comorbidities are summarized in Table 2. The majority of patients were men (59% of the symptomatic subgroup and 67% of the asymptomatic subgroup). Patients in the symptomatic subgroup were older than those in the asymptomatic subgroup (mean age: 62.8 years vs. 55.5 years) and had a higher Charlson Comorbidity Index score (mean: 2.82 vs. 1.69). The distribution of participants according to age group is summarized in Supplementary Fig. S3.

**Table 2:** Baseline characteristics, medical history, and comorbidities in the study population

Category	Total obstructive HCM	Asymptomatic	Symptomatic
	(*N* = 1141)	(*n* = 492)	(*n* = 649)
Sex, *n* (%)			
Male	711 (62.3)	331 (67.3)	380 (58.6)
Female	430 (37.7)	161 (32.7)	269 (41.4)
Age, mean [SD], years	59.6 [16.9]	55.5 [17.6]	62.8 [15.6]
Age, *n* (%), years			
18–39	144 (12.6)	92 (18.7)	52 (8.0)
40–59	405 (35.5)	190 (38.6)	215 (33.1)
≥ 60	592 (51.9)	210 (42.7)	382 (58.9)
CCI score, mean [SD]	2.34 [2.28]	1.69 [2.03]	2.82 [2.35]
CCI score, *n* (%)			
0	234 (20.5)	163 (33.1)	71 (10.9)
1	302 (26.5)	139 (28.3)	163 (25.1)
2	201 (17.6)	81 (16.5)	120 (18.5)
3	119 (10.4)	31 (6.3)	88 (13.6)
≥ 4	285 (25.0)	78 (15.9)	207 (31.9)
Comorbidities in the baseline period, *n* (%)			
Hypertension	727 (63.7)	262 (53.3)	465 (71.6)
Hyperlipidemia	397 (34.8)	115 (23.4)	282 (43.5)
Coronary artery disease	285 (25.0)	67 (13.6)	218 (33.6)
Heart failure	246 (21.6)	32 (6.5)	214 (33.0)
Hypercholesterolemia	221 (19.4)	69 (14.0)	152 (23.4)
Diabetes, type II	213 (18.7)	67 (13.6)	146 (22.5)
Atrial fibrillation/flutter	172 (15.1)	35 (7.1)	137 (21.1)
Depression	160 (14.0)	47 (9.6)	113 (17.4)
COPD	150 (13.1)	43 (8.7)	107 (16.5)
Previous or current pacemaker, CRT, or implantable cardioverter–defibrillator	132 (11.6)	29 (5.9)	103 (15.9)
Asthma	110 (9.6)	33 (6.7)	77 (11.9)
Stroke/TIA	102 (8.9)	27 (5.5)	75 (11.6)
Peripheral vascular disease	87 (7.6)	27 (5.5)	60 (9.2)
Myocardial infarction	50 (4.4)	10 (2.0)	40 (6.2)
Conduction disorders^a^	45 (3.9)	17 (3.5)	28 (4.3)
Deep vein thrombosis/pulmonary embolism	34 (3.0)	5 (1.0)	29 (4.5)
Diabetes, type I	29 (2.5)	9 (1.8)	20 (3.1)
SRT			
Alcohol septal ablation	9 (0.8)	2 (0.4)	7 (1.1)
Surgical myectomy	1 (0.1)	0 (0)	1 (0.2)
Cardiac arrest	4 (0.4)	0 (0)	4 (0.6)
Duration of follow-up, mean, years	4.66	1.48	4.50

Data are shown for the full obstructive HCM cohort (*N* = 1141) and the asymptomatic (*n* = 492) and symptomatic (*n* = 649) cohort assignments at index. Percentages presented are subject to rounding. ^a^Combined and each component of ICD-10-GM I45. *CCI*, Charlson Comorbidity Index; *COPD*, chronic obstructive pulmonary disease; *CRT*, cardiac resynchronization therapy; *HCM*, hypertrophic cardiomyopathy; *ICD-10-GM*, International Classification of Diseases, 10th Revision, Germany Modification; *SD* standard deviation; *SRT* septal reduction therapy; *TIA* transient ischemic attack

The most common comorbidities at baseline included hypertension (64%), hyperlipidemia (35%), coronary artery disease (25%), and heart failure (22%). These comorbidities were more common in the symptomatic subgroup than in the asymptomatic subgroup. A higher proportion of patients in the symptomatic subgroup than those in the asymptomatic subgroup had a current or previous pacemaker, cardiac resynchronization therapy, or implantable cardioverter–defibrillator device (16% vs. 6%; Table 2).

### Health outcomes, healthcare resource utilization, and costs during the follow-up period

The mean (standard deviation [SD]) duration of follow-up was 4.7 (2.5) years overall; for the symptomatic subgroup, it was 4.5 (2.6) years and, for the asymptomatic subgroup, it was 1.5 (2.1) years. In total, 1042 patients had symptomatic disease at some point during the follow-up period (Supplementary Fig. S1).

Health outcomes during the follow-up period are summarized in Table 3. The incidence of all-cause mortality was 0.05 per 100 patient-years in the total obstructive HCM cohort and was higher in the symptomatic subgroup (0.05 per 100 patient-years) than in the asymptomatic subgroup (0.02 per 100 patient-years). Regarding SRT, alcohol septal ablation procedures were more common than septal myectomy (4.5% vs. 0.6%) during the follow-up period. In the symptomatic subgroup, versus the asymptomatic subgroup, there were higher incidences of various incident cardiovascular conditions and events, including atrial fibrillation (0.10 vs. 0.09 per 100 patient-years), ischemic heart disease (0.21 vs. 0.18 per 100 patient-years), and heart failure (0.17 vs. 0.10 per 100 patient-years).

**Table 3:** Incident health outcomes during follow-up

Outcome	Total obstructive HCM (*N* = 1141)			Asymptomatic^a^ (*n* = 498)			Symptomatic (*n* = 1042)		
	Patients, *n* (%)	Time to first event, mean [SD], days	Incidence per 100 PY	Patients, *n* (%)	Time to first event, mean [SD], days	Incidence per 100 PY	Patients, *n* (%)	Time to first event, mean [SD], days	Incidence per 100 PY
All-cause mortality	244 (21.4)	887.7 [783.8]	0.05	14 (2.8)	288.5 [603.1]	0.02	230 (22.1)	888.5 [773.2]	0.05
Atrial fibrillation/flutter	358 (31.4)	460.9 [741.9]	0.09	62 (12.4)	137.3 [409.5]	0.09	343 (32.9)	450.4 [726.5]	0.10
Cardiac arrest	38 (3.3)	952.6 [896.0]	0.01	5 (1.0)	26.4 [20.9]	0.01	35 (3.4)	1079.0 [865.8]	0.01
Cardiac dysrhythmias	634 (55.6)	355.5 [634.8]	0.22	142 (28.5)	147.9 [431.7]	0.24	582 (55.9)	333.2 [596.0]	0.25
Conduction disorders	119 (10.4)	595.6 [805.1]	0.02	32 (6.4)	81.3 [310.1]	0.05	101 (9.7)	645.2 [766.2]	0.02
Deep vein thrombosis/pulmonary embolism	96 (8.4)	624.5 [714.9]	0.02	12 (2.4)	300.3 [460.1]	0.02	90 (8.6)	566.3 [692.9]	0.02
Dilated cardiomyopathy	172 (15.1)	428.1 [648.4]	0.04	20 (4.0)	92.4 [230.7]	0.03	161 (15.5)	409.6 [636.4]	0.04
Heart failure	520 (45.6)	417.0 [660.7]	0.15	67 (13.5)	84.9 [173.3]	0.10	491 (47.1)	406.0 [638.9]	0.17
Implantable device (pacemaker, CRT, implantable cardioverter–defibrillator)	265 (23.2)	295.1 [549.6]	0.06	49 (9.8)	80.4 [186.9]	0.07	255 (24.5)	287.5 [544.6]	0.07
Ischemic heart disease	553 (48.5)	293.2 [557.7]	0.18	117 (23.5)	122.7 [404.0]	0.18	526 (50.5)	277.0 [534.6]	0.21
Myocardial infarction	87 (7.6)	649.5 [809.5]	0.02	13 (2.6)	163.1 [527.8]	0.02	81 (7.8)	642.5 [800.0]	0.02
SRT									
Alcohol septal ablation	51 (4.5)	624.5 [687.8]	0.01	4 (0.8)	32.3 [28.0]	0.01	48 (4.6)	563.5 [602.7]	0.01
Surgical myectomy	7 (0.6)	457.6 [711.6]	0	1 (0.2)	0	0	6 (0.6)	533.8 [747.5]	0
Stroke/TIA	180 (15.8)	411.1 [639.0]	0.04	34 (6.8)	38.6 [91.4]	0.05	172 (16.5)	412.2 [617.5]	0.04

Data are shown for the full obstructive HCM cohort (*n* = 1141), the cohort of patients who had asymptomatic disease at some stage during follow-up (*n* = 498), and the cohort of patients who had symptomatic disease at some stage during follow-up (*n* = 1042). ^a^Patients could change between asymptomatic and symptomatic subgroups during the study period based on their NYHA functional class. *CRT*, cardiac resynchronization therapy; *HCM*, hypertrophic cardiomyopathy; *NYHA*, New York Heart Association; *PY*, patient-years; *SD*, standard deviation; *SRT*, septal reduction therapy; *TIA*, transient ischemic attack

Annualized healthcare resource utilization during the follow-up period is summarized in Table 4. Across all settings of care, resource utilization was generally higher for patients in the symptomatic subgroup than for those in the asymptomatic subgroup, as demonstrated by the higher mean numbers of outpatient visits (13.3 vs. 10.8 visits per patient-year) and all-cause inpatient visits (0.69 vs. 0.47 visits per patient-year). A higher proportion of patients in the symptomatic subgroup (57%) also had at least one all-cause emergency outpatient visit than of those in the asymptomatic subgroup (27%). Mean length of stay for all-cause inpatient visits was longer for patients in the symptomatic subgroup than for those in the asymptomatic subgroup (8.67 vs. 7.36 days).

**Table 4:** Annualized healthcare resource utilization

	Total obstructive HCM (*N* = 1141)				Asymptomatic^a^ (*n* = 498)				Symptomatic (*n* = 1042)			
	Number of visits per year	Number of visits per PY	Patients with ≥ 1 visit, *n* (%)	Number of visits per PY with visits	Number of visits per year	Number of visits per PY	Patients with ≥ 1 visit, *n* (%)	Number of visits per PY with visits	Number of visits per year	Number of visits per PY	Patients with ≥ 1 visit, *n* (%)	Number of visits per PY with visits
Any outpatient cases^b^	14,816	12.99	1130 (99.0)	12.99	5373	10.79	493 (99.0)	10.80	13,905	13.34	1031 (98.9)	13.37
GP	5111	4.48	1119 (98.1)	4.50	1734	3.48	475 (95.4)	3.54	4837	4.64	1020 (97.9)	4.67
Cardiologist	1080	0.95	782 (68.5)	1.29	343	0.69	246 (49.4)	1.14	1030	0.99	684 (65.6)	1.36
Other specialist	7043	6.17	1101 (96.5)	6.25	2833	5.69	375 (75.3)	6.13	6514	6.25	1004 (96.4)	6.34
HCM-related outpatient cases^b^	3778	3.31	1017 (89.1)	3.55	1102	2.21	426 (85.5)	2.36	3636	3.49	877 (84.2)	3.88
GP	2512	2.20	785 (68.8)	2.95	685	1.38	287 (57.6)	2.32	2434	2.34	714 (68.5)	3.09
Cardiologist	724	0.63	576 (50.5)	1.15	274	0.55	211 (42.4)	1.08	675	0.65	477 (45.8)	1.24
Other specialist	293	0.26	272 (23.8)	0.96	77	0.16	60 (12.0)	0.85	285	0.27	236 (22.6)	1.05
Outpatient emergency cases^b^												
All-cause	466	0.41	680 (59.6)	0.62	171	0.34	136 (27.3)	0.66	437	0.42	591 (56.7)	0.65
HCM-related	43	0.04	92 (8.1)	0.41	17	0.04	22 (4.4)	0.68	39	0.04	78 (7.5)	0.43
Inpatient visits												
All-cause	754	0.66	895 (78.4)	0.84	235	0.47	219 (44.0)	1.11	720	0.69	790 (75.8)	0.87
	Mean LOS = 8.54 days				Mean LOS = 7.36 days				Mean LOS = 8.67 days			
HCM-related	69	0.06	164 (14.4)	0.37	32	0.06	43 (8.6)	1.65	62	0.06	142 (13.6)	0.37
	Mean LOS = 5.29 days				Mean LOS = 5.49 days				Mean LOS = 5.26 days			

Data represent mean numbers of visits and are shown for the full obstructive HCM cohort (*N* = 1141), the cohort of patients who had asymptomatic disease at some stage during follow-up (*n* = 498), and the cohort of patients who had symptomatic disease at some stage during follow-up (*n* = 1042). ^a^Patients could change between asymptomatic and symptomatic subgroups during the study period based on their NYHA functional class. ^b^Professional cases, with ‘cases’ representing outpatient care from the same provider for the same reason within a quarter and may represent multiple visits. *GP*, general practitioner; *HCM*, hypertrophic cardiomyopathy; *LOS*, length of stay; *NYHA*, New York Heart Association; *PY*, patient-year

**Fig. 2:**
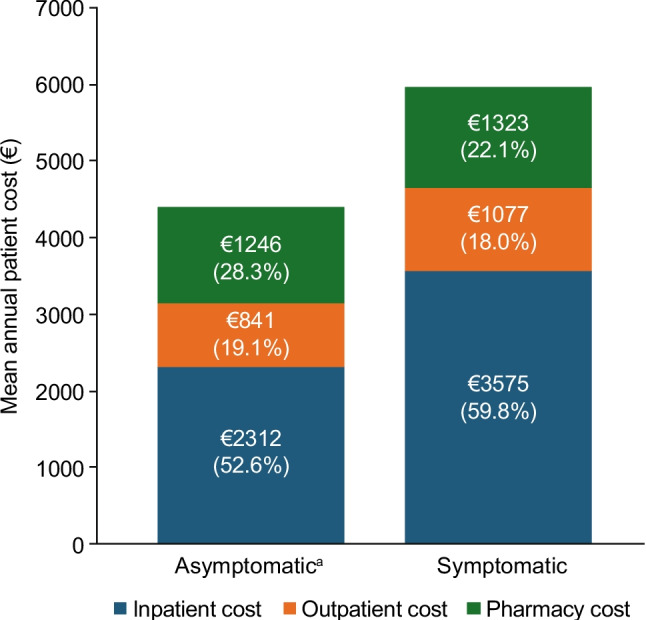
Mean annual patient costs. Data are shown for the cohort of patients who had asymptomatic disease at some stage during follow-up (*n* = 498) and the cohort of patients who had symptomatic disease at some stage during follow-up (*n* = 1042). Percentage values refer to the proportion of the mean total annualized costs per patient in each subgroup that relate to inpatient costs, outpatient costs and pharmacy costs. ^a^Patients could change between asymptomatic and symptomatic subgroups during the study period based on their NYHA functional class. *HCM* hypertrophic cardiomyopathy, *NYHA* New York Heart Association

Annualized costs per patient are presented in Fig. 2**.** The total mean cost per patient per year for outpatient, inpatient and pharmacy services combined was €5975 in the symptomatic subgroup versus €4399 in the asymptomatic subgroup. Costs (mean [SD]) per patient per year were also higher for patients in the symptomatic subgroup than for those in the asymptomatic subgroup for outpatient services (€1077 [€2240] vs. €841 [€1981]), inpatient services (€3575 [€9785] vs. €2312 [€12,651]), and pharmacy services (€1323 [€3245] vs. €1246 [€4784]). In both asymptomatic and symptomatic subgroups, costs related to inpatient services accounted for the highest proportion of the mean total annualized costs per patient (53% and 60%, respectively), followed by costs related to pharmacy services (28% and 22%) and outpatient services (19% and 18%).

## Discussion

This is the first study to provide a detailed analysis of the clinical and economic burden associated with symptomatic and asymptomatic obstructive HCM in Germany. Overall, the results demonstrated that the annual prevalence of obstructive HCM increased over time from 2011 to 2019. Moreover, patients with symptomatic obstructive HCM experienced a greater burden than those with asymptomatic disease, as demonstrated by higher all-cause mortality, higher incidences of cardiovascular conditions and events, higher healthcare resource utilization, and higher average annual overall costs across all settings and types of care.

The estimated annual prevalence of obstructive HCM in the present study is in alignment with a previous study in Germany, which reported an HCM prevalence of approximately 0.07% based on claims data in 2015 [8]. Although these estimates are lower than previously established prevalence estimates of 1:500 (0.2%) for HCM, as reported by Maron et al. [4], this difference likely reflects the identification of cases from claims data, rather than identification of HCM diagnoses based on population screening by echocardiography. Furthermore, in alignment with the previous analysis in Germany, annual prevalence increased year-on-year [8]. Interestingly, this linear trend was observed in both the symptomatic and asymptomatic subgroups, indicating that this increase is not merely driven by classification of one type over another but is likely showing a true increase in the number of HCM diagnoses.

During follow-up, a substantial proportion of participants experienced incident cardiovascular events. Given that previous studies have reported associations between worsening NYHA functional class and increased risks of cardiovascular events in the USA [12], this may indicate an unmet need for symptom management in these patients.

The findings of this study in Germany build on previous research that reported substantial healthcare resource utilization and costs in patients with obstructive HCM compared with matched controls in the USA [13]. As expected, the overall burden was higher in patients with symptomatic obstructive HCM than in those with asymptomatic disease, which may suggest a relationship between symptom levels and resource utilization. Indeed, in patients with symptomatic obstructive HCM, compared with those with asymptomatic disease, the average number of annual visits was greater for both all-cause and HCM-related outpatient visits. Furthermore, annual costs were also higher in the symptomatic subgroup than in the asymptomatic subgroup for outpatient, inpatient, and pharmacy services. The largest between-subgroup difference was found for inpatient costs. As such, this suggests that the overall increased cost among patients with a higher symptom burden is largely driven by increased costs associated with inpatient services. Regardless, these findings should also be considered in the context of the differences in baseline characteristics, which may have affected resource utilization. For example, compared with those in the asymptomatic subgroup, patients in the symptomatic subgroup were older, had longer follow-up times on average, and had higher rates of comorbidities such as atrial fibrillation and hypertension.

Future studies should investigate whether treatments for symptomatic obstructive HCM, such as cardiac myosin inhibitors, can reduce overall symptom burden, decrease healthcare utilization, and improve outcomes among eligible patients. Other avenues of research could also look further into the increasing prevalence of obstructive HCM in the German population and could assess if similar increases are being observed elsewhere.

### Limitations

This study was based on German SHI data (secondary data) intended for the reimbursement of healthcare costs. The quality of claims data is dependent on the individual completeness and quality of coding for billing purposes and on the existing classification systems; therefore, this study has all the limitations of similar studies based on administrative data. Furthermore, use of SHI data meant that the clinical and economic burden of obstructive HCM in German patients with private health insurance was not assessed in this study. NYHA functional class and symptoms are rarely coded in the database and were assigned to subgroups based on an algorithm developed with clinical expert input. This algorithm should be considered to be a proxy for actual NYHA functional class, but it is possible that some patients with symptomatic disease were incorrectly assigned as having asymptomatic disease in this study, and vice versa. Further research is needed to validate this algorithm fully; potential methods to assess the algorithm include analyzing its accuracy in identifying NYHA functional class in clinical registries or prospective studies where this information is recorded. Exact dates cannot be determined for outpatient diagnoses in this dataset; therefore, the index date was set to the first day of the quarter. The determination of the number of outpatient visits within a quarter was also subject to this restriction. Finally, some patients who were classified as having asymptomatic and symptomatic disease at the index date changed categories during the follow-up period, meaning that comparisons between groups were not truly independent.

## Conclusions

In conclusion, healthcare resource utilization and costs associated with obstructive HCM are substantially higher for patients with symptomatic disease than for those with asymptomatic disease in Germany.

## Supplementary Information

Below is the link to the electronic supplementary material.

ESM1 (DOCX. 348 KB)

## Data Availability

Bristol Myers Squibb’s policy on data sharing may be found at: https://www.bms.com/researchers-and-partners/independent-research/data-sharing-request-process.html
