# Supplementary material for: Clinical and economic burden associated with symptomatic and asymptomatic obstructive hypertrophic cardiomyopathy in Germany
Source: Clin Res Cardiol. 2025 Oct 27;115(5):852–61. doi: 10.1007/s00392-025-02776-4 (PMC13083345; doi:10.1007/s00392-025-02776-4)
Supplement: Supplementary file 1 — (DOCX. 348 KB) [file 392_2025_2776_MOESM1_ESM.docx]

*Original Article*

Clinical and economic burden associated with symptomatic and asymptomatic obstructive hypertrophic cardiomyopathy in Germany

Farbod Sedaghat-Hamedani^1,2^ · Carla L. Zema^3^* · Michael Schultze^4^ · Tarcyane B. Garcia^5^ · Nils Kossack^5^ · Julia Borchert^5^ · Ervant J. Maksabedian Hernandez^3^ · Yue Zhong^3^ · Tobias Bluhmki^6^ · Taryn Krause^7^* · Johanna Schmoelders^6^ · Benjamin Meder^1,2^

^1^Department of Internal Medicine III, Institute of Cardiomyopathies, University of Heidelberg, Heidelberg, Germany

^2^DZHK (German Centre for Cardiovascular Research), Heidelberg, Germany

^3^Bristol Myers Squibb, Princeton, NJ, USA

^4^ZEG – Berlin Center for Epidemiology and Health Research GmbH, Berlin, Germany

^5^WIG2 GmbH – Scientific Institute for Health Economics and Health System Research, Leipzig, Germany

^6^Bristol Myers Squibb, Munich, Germany

^7^Bristol Myers Squibb, Uxbridge, UK

*Affiliation at the time the analysis was conducted.

Correspondence: Professor Benjamin Meder

Department of Medicine III

University of Heidelberg

INF 410, 69120 Heidelberg

Germany
e-mail: benjamin.meder@med.uni-heidelberg.de

SUPPLEMENTARY MATERIALS


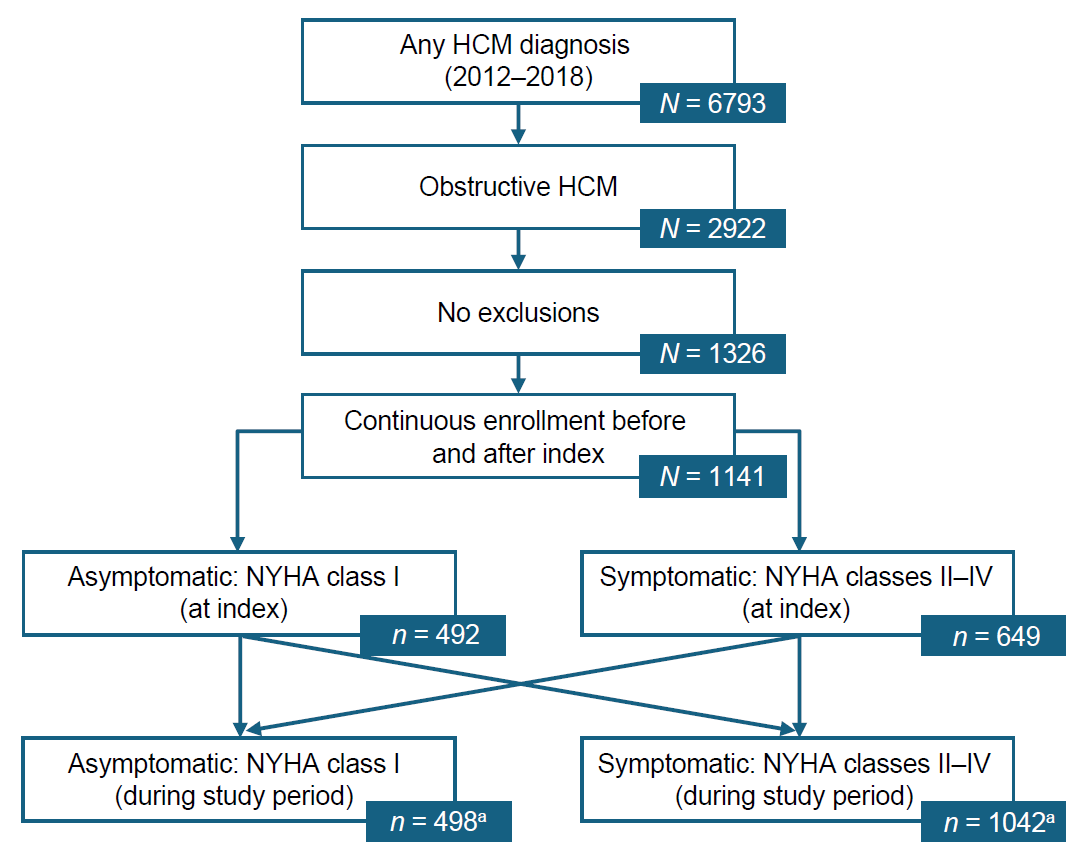


**Supplementary Fig. S1** Study population and sample selection. ^a^Patients could change between asymptomatic and symptomatic subgroups during the study period based on their NYHA functional class. HCM hypertrophic cardiomyopathy, NYHA New York Heart Association


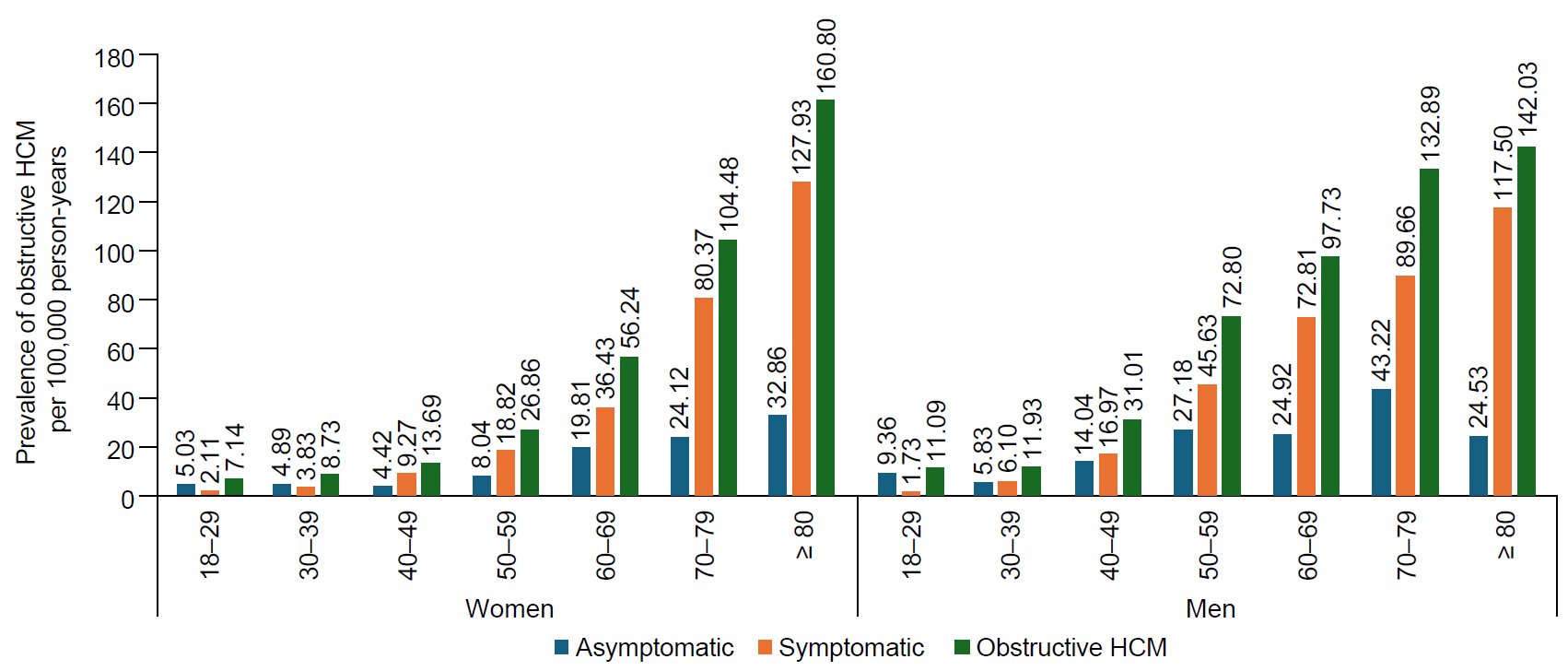


**Supplementary Fig. S2** Prevalence of obstructive HCM in the SHI population in 2019. Prevalence of obstructive HCM per 100,000 person-years in the SHI population in 2019 is stratified by symptomatic/asymptomatic subgroups, sex, and age groups. HCM hypertrophic cardiomyopathy, SHI statutory health insurance


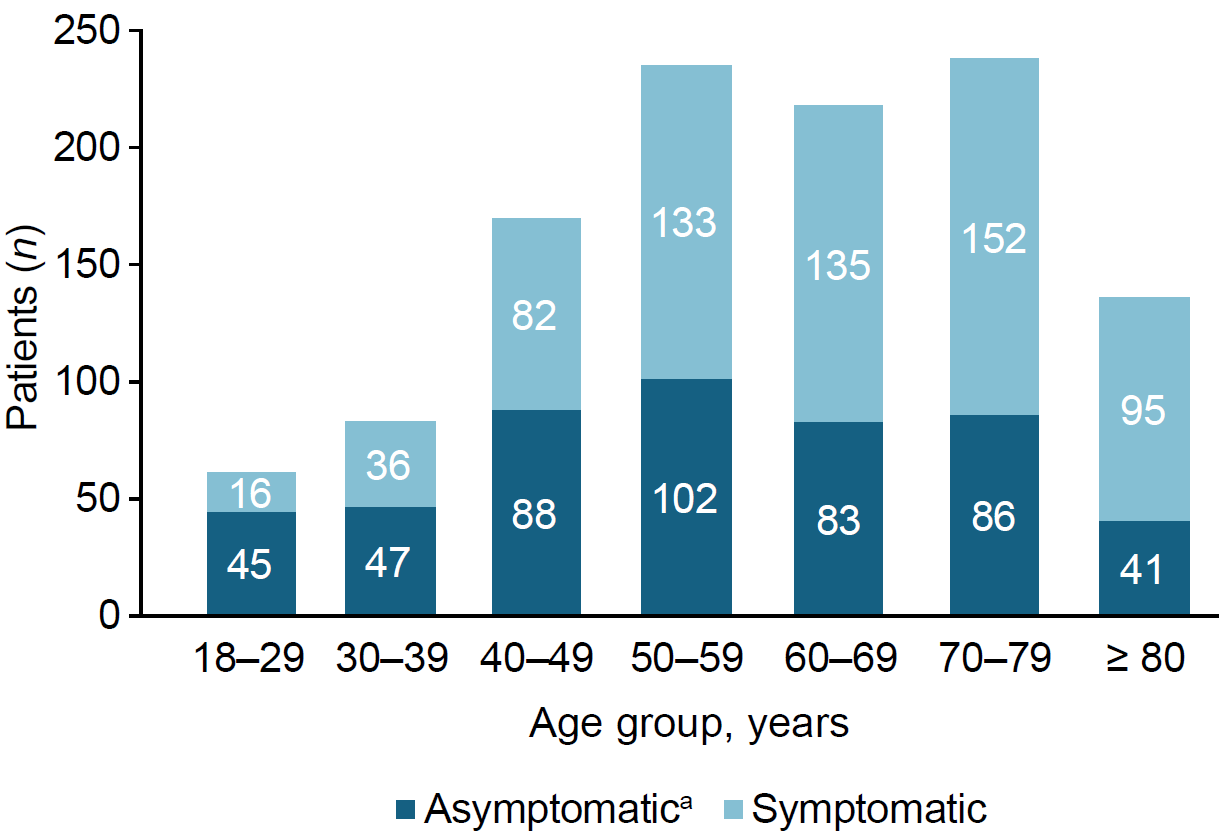


**Supplementary Fig. S3** Histogram of patients’ ages at baseline. ^a^Patients could change between asymptomatic and symptomatic subgroups during the study period based on their NYHA functional class. *NYHA* New York Heart Association
